# EDLm^6^APred: ensemble deep learning approach for mRNA m^6^A site prediction

**DOI:** 10.1186/s12859-021-04206-4

**Published:** 2021-05-29

**Authors:** Lin Zhang, Gangshen Li, Xiuyu Li, Honglei Wang, Shutao Chen, Hui Liu

**Affiliations:** 1 Engineering Research Center of Intelligent Control for Underground Space, Ministry of Education, China University of Mining and Technology, Xuzhou, 221116 China; 2 School of Information and Control Engineering, China University of Mining and Technology, Xuzhou, 221116 China

**Keywords:** m^6^A methylation modification, Word embedding, Deep learning, Predictor

## Abstract

**Background:**

As a common and abundant RNA methylation modification, N6-methyladenosine (m^6^A) is widely spread in various species' transcriptomes, and it is closely related to the occurrence and development of various life processes and diseases. Thus, accurate identification of m^6^A methylation sites has become a hot topic. Most biological methods rely on high-throughput sequencing technology, which places great demands on the sequencing library preparation and data analysis. Thus, various machine learning methods have been proposed to extract various types of features based on sequences, then occupied conventional classifiers, such as SVM, RF, etc., for m^6^A methylation site identification. However, the identification performance relies heavily on the extracted features, which still need to be improved.

**Results:**

This paper mainly studies feature extraction and classification of m^6^A methylation sites in a natural language processing way, which manages to organically integrate the feature extraction and classification simultaneously, with consideration of upstream and downstream information of m^6^A sites. One-hot, RNA word embedding, and Word2vec are adopted to depict sites from the perspectives of the base as well as its upstream and downstream sequence. The BiLSTM model, a well-known sequence model, was then constructed to discriminate the sequences with potential m^6^A sites. Since the above-mentioned three feature extraction methods focus on different perspectives of m^6^A sites, an ensemble deep learning predictor (EDLm^6^APred) was finally constructed for m^6^A site prediction. Experimental results on human and mouse data sets show that EDLm^6^APred outperforms the other single ones, indicating that base, upstream, and downstream information are all essential for m^6^A site detection. Compared with the existing m^6^A methylation site prediction models without genomic features, EDLm^6^APred obtains 86.6% of the area under receiver operating curve on the human data sets, indicating the effectiveness of sequential modeling on RNA. To maximize user convenience, a webserver was developed as an implementation of EDLm^6^APred and made publicly available at www.xjtlu.edu.cn/biologicalsciences/EDLm6APred.

**Conclusions:**

Our proposed EDLm^6^APred method is a reliable predictor for m^6^A methylation sites.

## Background

N6-methyladenosine (m^6^A) methylation modification refers to the methylation that occurs on the sixth N atom of base A [1], accounting for 80% of eukaryotic mRNA methylation modifications [2, 3]. It was first discovered in the 1970s [4] and has been found to exist in many species such as animals, plants, bacteria, viruses [5]. All sites were found within sequences conforming to the degenerate consensus RRACH(A = m^6^A) [6, 7]. Studies have found that m^6^A plays a crucial role in various biological processes and ontogeny, including mRNA transcription, translation, nucleation, splicing, and degradation [8], as well as early development, sex determination, T cell homeostasis, antiviral immunity, brain development, biological rhythms, sperm genesis and directed differentiation of hematopoietic stem cells [9–13]. Besides, m^6^A methylation modification has been found to play a key role in the occurrence of diseases, such as glioma, leukemia, hepatocellular carcinoma, etc., [14–16]. Therefore, it is of great significance to unveil the mechanism of m^6^A methylation, where the specific modification sites should be first identified accurately.

At present, high-throughput sequencing technologies are widely used in the study of m^6^A modification, among which MeRIP-Seq is the most commonly used [17]. The procedure for MeRIP-Seq involves randomly fragmenting the RNA to fragments (namely reads) before immunoprecipitation, these reads are expected to map to a region that contains the m^6^A site near its center. Reads from the immunoprecipitation sample are frequently mapped to mRNAs and clustered as distinct peaks [18, 19]. Experimental-based high-throughput sequencing methods can perform sample-specific m^6^A site detection [20]. However, the MeRIP-Seq technology is relatively complicated with high cost and time, which limits its extensive use. Thus, some computational methods that can help predict m^6^A modification sites computationally are urgently needed.

Most conventional machine learning methods developed for sequence-based m^6^A site prediction often extract features first, then, developed classifiers to predict whether a site is methylated or not based on previously extracted features. For example, iRNA-Methyl extracts features based on pseudo dinucleotide composition, three RNA physiochemical properties, and uses SVM to construct a site prediction model [21]. SRAMP extracts features with three encoding methods, including positional binary encoding of nucleotide sequence, K-nearest neighbor (KNN) encoding as well as nucleotide pair spectrum encoding, then predicts sites by random forest classifiers respectively. Finally, the prediction scores of the random forest classifiers are combined through the weighted summing formula [22]. AthMethPre extracts the features of the positional flanking nucleotide sequence and position-independent k-mer nucleotide spectrum then uses an SVM classifier to predict m^6^A methylation sites [23]. The WHISTLE method firstly integrates 35 additional genomic features besides the conventional sequence features and then establishes an SVM classifier to predict m^6^A sites [24]. The prediction performance was greatly improved through the use of genomic features. However, genomic features are not always available under the scenarios that only some RNA sequences are given for m^6^A site identification. It is shown that the extraction of RNA sequence features and the design of classifiers all have an impact on the prediction performance of m^6^A modification sites. The methods mentioned above all establish a closed feature extraction model, which is independent of the following classifiers. Feature extraction is the key issue for most machine learning tasks. The quality of feature extraction is extremely critical. which greatly affects the performance of the final site prediction. On the contrary, deep learning models often follow the end-to-end design. From raw data to final output, the features are extracted based on both the input data and the final identification/prediction task. Besides, considering that RNA sequence contains abundant semantic information, which is similar to text sequences, it is heuristic that some text sequence representation methods developed in the field of NLP (Natural Language Processing) may apply to the RNA sequence. To be more specific, Gene2vec uses Word2vec [25] and Convolutional Neural Network (CNN) to predict m^6^A sites [26]. DeepPromise uses ENAC, One-hot [27], and RNA embedding [28] to achieve feature encoding of RNA sequences, and then integrates CNN model scores to achieve m^6^A site prediction [29]. By integrating BGRU with word embedding and a Random Forest classifier with a novel encoding of enhanced nucleic acid content (ENAC), BERMP can better identify m6A sites, which demonstrates that the deep learning framework is more suitable for addressing the prediction task with larger datasets [30]. However, the prediction performance of existing methods can still be improved. Thus, this paper further proposes an ensemble deep learning m^6^A site predictor EDLm^6^APred based on a recurrent neural network framework. It uses three encoding methods, including One-hot, RNA word embedding as well as Word2vec to depict RNA sequences. Based on the vectorized sequence representation obtained by the above-mentioned encoding methods, bi-directional long short-term memory (BiLSTM) is then constructed to achieve feature extraction and site prediction simultaneously. Finally, the prediction of m^6^A modification sites was completed by weighted integration of three prediction scores figured by the BiLSTM model trained with three different feature encodings. Fivefold cross-validation experiments on 3 independent test sets were conducted, with metrics such as the area under the ROC curve (AUROC), accuracy (ACC), precision (Precision), recall (Recall), and Matthews correlation coefficient (MCC) were calculated to compare with the performance of state-of-the-art methods such as Gene2vec and DeepPromise.

## Results

### Performance evaluation

In this paper, we adopted widely used evaluation indexes to evaluate the performance of EDLm^6^APred, including Area Under the Receiver Operation Curve (AUROC), Precision, Recall, Accuracy (ACC), and the Matthews correlation coefficient (MCC). These are the most widely used metrics for binary classifier evaluation, and the definition of ACC, Precision, Recall, and MCC are given in (1–4) [31, 32].

1 $$precision = \frac{TP}{{TP + FP}}$$

2 $$Recall = \frac{TP}{{TP + FN}}$$

3 $$Acc = \frac{TP + TN}{{TP + TN + FP + FN}}$$

4 $$MCC = \frac{TP \times TN - FP \times FN}{{\sqrt {(TP + FP) \times (TP + FN) \times (TN + FP) \times (TN + FN)} }}$$

where *TP* refers to true positives, counting the number of positive samples that are truly predicted as positive. *TN* refers to true negatives, indicating the number of correctly classified negative samples. *FP* refers to false positives, which is the number of negative samples that are incorrectly classified as positive. *FN* is false negatives, which refers to the number of positive samples that are incorrectly classified as negative.

### Results analysis

In this paper, we first evaluated the effect of different sequence pre-processing methods, different sequence representation methods, and commonly used deep learning models on the prediction results respectively. Then, we evaluated the performance of the EDLm^6^APred predictor. Finally, we also compared our method with the newest predictor of m^6^A sites.

First, we tested three different sequence pre-processing methods based on human data set to compare their impact on model performance, which are overlapping equal length, overlapping variable length, and non-overlapping equal length. For example, given a sequence:

$${\text{AGGTCAGCATGC}}$$

In the processing of overlapping equal-length, a sliding window of size 3nt was used to slide on the sequence with one stride. Finally, we obtained a series of sub-sequences composed of 3 bases. The processing result of the above hypothetical sequence is as follows:

$${\text{AGG}}\;{\text{GGT}}\;{\text{GTC}}\;{\text{TCA}}\;{\text{CAG}}\;{\text{AGC}}\;{\text{GCA}}\;{\text{CAT}}\;{\text{ATG}}\;{\text{TGC}}$$

In the processing of overlapping variable length, K was sampled from the discrete uniform distribution Uniform (K_low_, K_high_) to determine each window’s size. In this paper, we set K_low_ = 3 and K_high_ = 5. The processing result of the above hypothetical sequence is as follows:

$${\text{AGG}}\;{\text{GGTC}}\;{\text{GTC}}\;{\text{TCAGC}}\;{\text{CAG}}\;{\text{AGCA}}\;{\text{GCAT}}\;{\text{CATGC}}$$

In the processing of non-overlapping equal length, a sliding window of size 3nt was used to slide on the sequence with three strides. Finally, we obtained a series of sub-sequences composed of 3 bases. The processing result of the above hypothetical sequence is as follows:

$${\text{AGG}}\;{\text{TCA}}\;{\text{GCA}}\;{\text{TGC}}$$

**Fig. 1:**
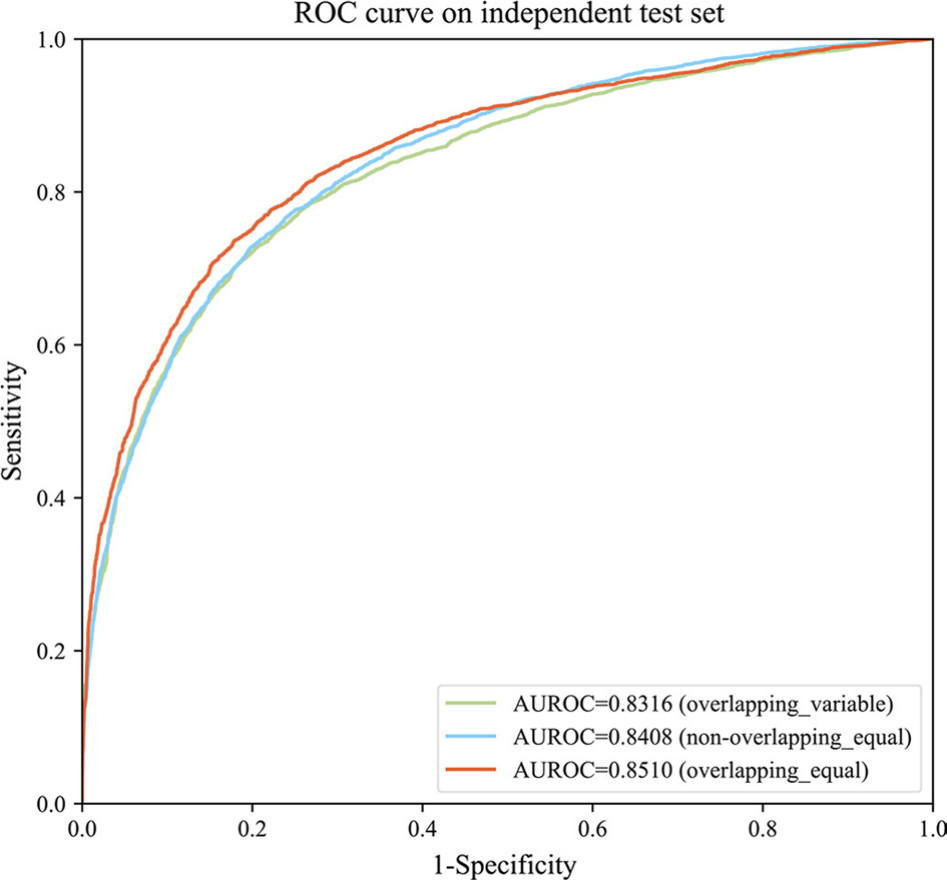
ROC curves of different sequence pre-processing methods on the human independent test set. The sequence pre-processing methods are overlapping equal length, overlapping variable length, and non-overlapping equal length respectively

After pre-processing, all the sub-sequences produced by the above three methods are fed into the Word2vec based predictor for further site identification. The ROC curves are shown in Fig. 1. It shows that the performance of prediction with overlapping equal length method is better than the others. Therefore, the overlapping equal length method was used to complete the sequence pre-processing in the following experiments.

**Fig. 2:**
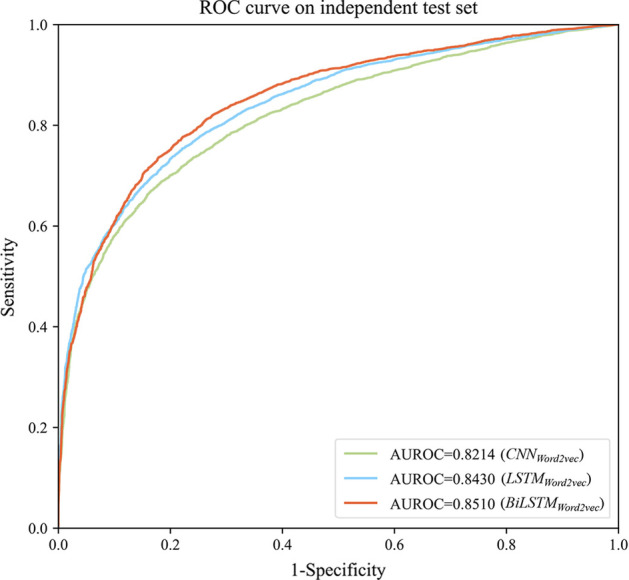
ROC curves of different deep learning models on the human independent test set. The deep learning models are CNN, LSTM, and BiLSTM respectively

Next, the BiLSTM model has been compared with the LSTM and CNN models. This group of experiments adopted Word2vec to represent sequences, which were denoted as CNN_Word2vec_, LSTM_Word2vec_, and BiLSTM_Word2vec_ respectively. The evaluation results and ROC curves of the fivefold cross-validation on the human data set are shown in Table 1 and Fig. 2. It shows that the AUROC of the three models from high to low is BiLSTM_Word2vec_, LSTM_Word2vec_, and CNN_Word2vec_. LSTM_Word2vec_ is nearly 2% higher than CNN_Word2vec_, and BiLSTM_Word2vec_ is nearly 1% higher than LSTM_Word2vec_. The reason may be that the essence of CNN is to extract the local features of the sequence while ignoring the context. However, the LSTM and BiLSTM model based on RNN can better capture the interaction between distant elements in the sequence and obtain the relative position relation between each sub-sequence. Thus, they can extract the global features of the sequence. Besides, the pooling layer after CNN may lead to the loss of important location information. In addition, BiLSTM performs better than LSTM, possibly because BiLSTM is composed of forward LSTM and backward LSTM, which can capture context information simultaneously, while one-way LSTM may capture upstream or downstream information only.

**Table 1:** Evaluation results of different deep learning models

Classifiers	AUROC	MCC	ACC	Precision	Recall
CNN_Word2vec_	0.8214	0.5098	0.7458	0.8348	0.6102
LSTM_Word2vec_	0.8430	0.5368	0.7650	0.8155	0.6821
BiLSTM_Word2vec_	**0.8510**	**0.5497**	**0.7695**	**0.8361**	0.6678

The evaluation indexes with BiLSTM better than LSTM and CNN are show in bold

**Fig. 3:**
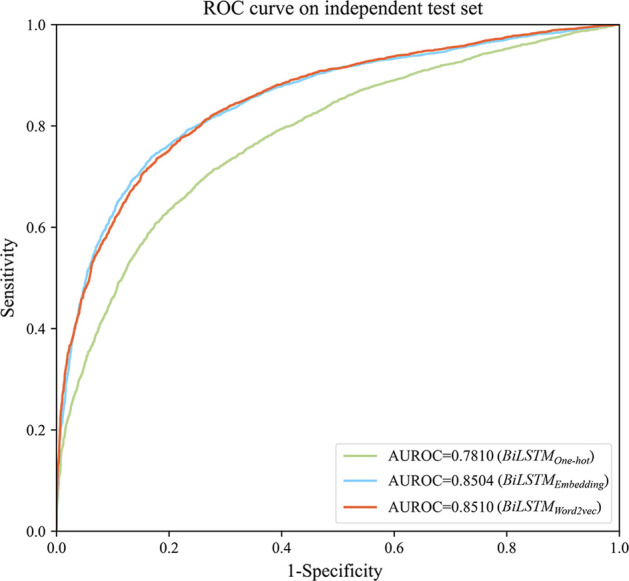
ROC curves of different sequence encoding modes on the human independent test set. The sequence encoding modes are One-hot, RNA word embedding, and Word2vec respectively

Besides, the prediction performance of the three different feature encoding methods was compared. This group of experiments firstly encoded the sequences by One-hot, RNA word embedding, and Word2vec respectively, then adopted the same BiLSTM classifier framework for further site identification. The performance all went through the above procedure. A fivefold cross-validation experiment was carried out on the human data set. The ROC curves on the independent test set are shown in Fig. 3. It can be seen that the AUROC of Word2vec based model achieves 0.8510, which is higher than RNA word embedding and One-hot based ones.

These three encoding methods represent sequences from different perspectives. In this paper, a deep prediction model EDLm^6^APred was constructed to perform weighted integration of the three predictors. fivefold cross-validation experiments were conducted on the human data set, mouse data set, and mixed data set of human and mouse respectively. The results in the independent test set are shown in Table 2. All the performance of EDLm^6^APred is superior to any single predictor. The integration of the three predictors not only considers the location information of the sequence but also considers its context information, which achieves the complementary advantages.

**Table 2:** Evaluation results of single predictor and integrated predictor based on different species

Species	Classifiers	AUROC	MCC	ACC	Precision	Recall
Human	BiLSTM_One-hot_	0.7810	0.4409	0.7159	0.7716	0.6095
	BiLSTM_Embedding_	0.8504	0.5602	0.7739	0.8470	0.6661
	BiLSTM_Word2vec_	0.8510	0.5497	0.7695	0.8361	0.6678
	EDLm^6^APred	**0.8660**	**0.5819**	**0.7843**	**0.8617**	**0.6750**
Mouse	BiLSTM_One-hot_	0.7838	0.4354	0.7088	0.7901	0.5739
	BiLSTM_Embedding_	0.8390	0.5394	0.7642	0.8296	0.6691
	BiLSTM_Word2vec_	0.8464	0.5369	0.7604	0.8429	0.6442
	EDLm^6^APred	**0.8588**	**0.5664**	**0.7754**	**0.8579**	0.6639
Mix	BiLSTM_One-hot_	0.8055	0.4758	0.7361	0.7687	0.6755
	BiLSTM_Embedding_	0.8459	0.5670	0.7801	0.8313	0.7028
	BiLSTM_Word2vec_	0.8463	0.5477	0.7707	0.8189	0.6952
	EDLm^6^APred	**0.8605**	**0.5787**	**0.7862**	**0.8355**	**0.7128**

The evaluation indexes with EDLm^6^ APred better than its any single predictor are show in bold

This paper compared EDLm^6^APred with DeepPromise. We replaced the CNN model in DeepPromise with BiLSTM to construct BiLSTM_DeepPromise_ and replaced the ENAC encoding in BiLSTM_DeepPromise_ with Word2vec to construct our EDLm^6^APred predictor. Fivefold cross-validation experiments were conducted on the human data set, mouse data set, and mixed data set of human and mouse respectively. The results are shown in Table 3.

**Table 3:** Compare with DeepPromise predictors

Species	Classifiers	AUROC	MCC	ACC	Precision	Recall
Human	DeepPromise	0.8302	0.5164	0.7576	0.7769	0.7196
	BiLSTM_DeepPromise_	0.8592	0.5707	0.7780	0.8593	0.6626
	EDLm^6^APred	**0.8660**	**0.5819**	**0.7843**	**0.8617**	0.6750
Mouse	DeepPromise	0.8381	0.5242	0.7613	0.7832	0.7272
	BiLSTM_DeepPromise_	0.8524	0.5625	0.7760	0.8409	0.6847
	EDLm^6^APred	**0.8588**	**0.5664**	0.7754	**0.8579**	0.6639
Mix	DeepPromise	0.8348	0.5208	0.7599	0.7766	0.7298
	BiLSTM_DeepPromise_	0.8546	0.5748	0.7840	0.8354	0.7075
	EDLm^6^APred	**0.8605**	**0.5787**	**0.7862**	**0.8355**	0.7128

The evaluation indexes with EDLm^6^ APred better than DeepPromise are show in bold

The AUROC of EDLm^6^APred is significantly better than DeepPromise and BiLSTM_DeepPromise_. Since ENAC encoding only considers the nucleic acid composition and position information of the sequence but fails to consider the more in-depth semantic information of the sequence, while Word2vec can better represent the sequence. In addition, BiLSTM is more suitable to capture the features of the RNA sequence than CNN.

## Discussion

In this paper, the m^6^A site predictor EDLm^6^APred was constructed based on the word embedding algorithm and Bi-directional Long Short-Term Memory Recurrent Neural Network to explore various RNA sequence pre-processing and feature encoding methods. We compared Three data pre-processing methods, including overlapping equal length, overlapping variable length, and non-overlapping equal length. Finally, the overlapping equal length method was selected to complete the pre-processing of the RNA sequence. Then, we obtained the feature representation of the sequence by three encoding methods of One-hot, RNA word embedding, and Word2vec. Moreover, we compared the effect of three deep learning models respectively on the site prediction performance, including CNN, LSTM as well as BiLSTM. The experimental results showed that the BiLSTM model can significantly improve the prediction performance. Considering that different encoding approaches depict the sequence from different perspectives, which may be complementary to each other, EDLm^6^APred combined the former mentioned encoding methods followed by the BiLSTM model together with weights to obtain the final prediction.

## Conclusions

The contribution of this paper lies in the proposition of an m^6^A site predictor EDLm^6^APred under a deep recurrent neural network framework. In this paper, different RNA sequence feature encoding methods were employed to decipher RNA sequences more thoroughly, and the BiLSTM model was employed to better take advantage of contextual information for m^6^A site prediction.

## Methods

### Data and its sequence representation

This paper is based on the two sets of human and mouse data sets established by Zou et al. Both data sets obtained complementary DNA (cDNA) sequence data from the Ensemble database [33]. After obtaining mRNA sequences through reverse complementation, sequences that were not GAC or AAC motif in the center were removed, and sequences shorter than 1001nt were filled with the character “X”. Finally, the sequences of the data sets used for algorithm training are 1001nt, and the proportion of positive and negative samples is 1:1. See the data sets on the webserver www.xjtlu.edu.cn/biologicalsciences/EDLm6APred for details.

The effective feature encoding method determines the performance of the site prediction model. The sequences are first encoded in the way of one-hot, RNA word embedding, and Word2vec respectively. One-hot and RNA word embedding are standard approaches for RNA sequence encoding. High-dimensional sparse binary word vector and low-dimensional dense word vector are obtained to characterize RNA modification sites. Word2vec can effectively extract relevant semantic features according to the upstream and downstream context of the base, then translate them into word vector expression.

5-dimensional binary vectors are introduced as one-hot encoding to represent each single base in the RNA sequence, corresponding to four nucleotides and the filling character “X” respectively. To be specific, A = [1, 0, 0, 0, 0], T = [0, 1, 0, 0, 0], G = [0, 0, 1, 0, 0], C = [0, 0, 0, 1, 0] and X = [0, 0, 0, 0, 1]. Therefore, each sequence of 1001 bps in the dataset is converted to binary vectors of 5005 bits.

Following the idea of RNA Word embedding coding, a 3nt window is used to slide over for each sequence to obtain 999 sub-sequences composed of 3 bases. Finally, 105 different sub-sequences and the unique integer indexes corresponding to the 105 sub-sequences in the dictionary are obtained. A unique integer index represents the pseudo-RNA word, and each pre-processed sequence is converted into an integer sequence with a corresponding integer index, then fed into the embedding layer. Therefore, a sequence of 1001nts in the dataset is converted into a matrix of 999 × 100, where 100 is the dimension of the word vector.

Word2vec encoding can be achieved following CBOW or Skip-gram models. The CBOW model is usually used to predict the current word based on its context, while the Skip-gram model predicts the context based on the current word. CBOW model is known to run faster than the skip-gram model in training. Besides, the number of data sets used in our experiment is relatively large, and the types of words in the corpus are small (105 types). There are no uncommon words and words with low frequency. Thus, the CBOW model is followed to encode RNA sequences in this paper. To be more specific, the sequences are first divided into sub-sequences of length 3nt by overlapping equal length, then the CBOW model is used for training. Therefore, each sub-sequence is transformed to represent the semantic word vector, and then the obtained word vector is used to represent the sequence of 1001nt in the data set into a matrix of 999 × 100. The input and output of these encoding methods are shown in Table 4.

**Table 4:** Three feature encoding input and output formats

Encoding	Input	Output
One-hot	1nt sequences of length 1001	Binary vectors of length 5005
RNA word embedding	3nt sequences of length 999	Matrix (999 × 100)
Word2vec	3nt sequences of length 999	Word vectors with dimension 100

### BiLSTM

**Fig. 4:**
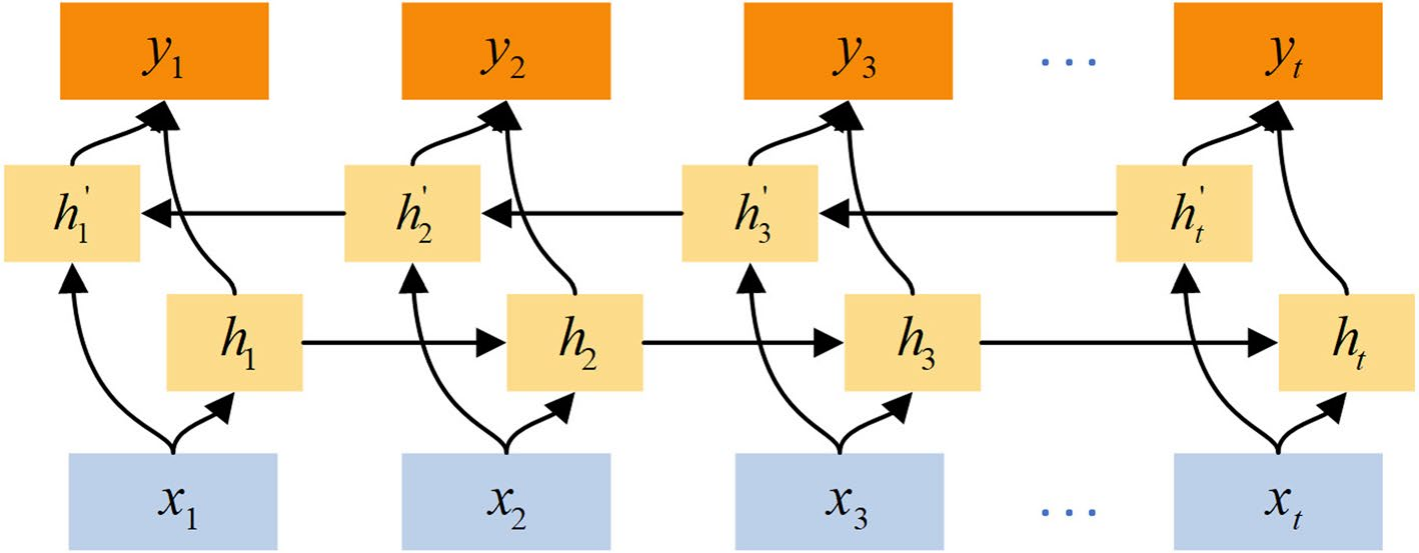
BiLSTM model diagram, which consists of the forward LSTM layer and the backward LSTM layer

BiLSTM is developed from RNN (Recurrent Neural Network) and consists of two parts, the forward LSTM(Long Short-Term Memory) layer and the backward LSTM layer [34, 35]. Its structure is shown in Fig. 4. The forward calculation is performed from moment 1 to moment *t* in the forward LSTM layer to obtain and save the forward hidden layer's output at each moment. At the same time, the backward calculation is performed from moment *t* to moment 1 in the backward LSTM layer to obtain and save the backward hidden layer's output at each moment. Finally, the final output is obtained at each moment by combining the output results of the forward LSTM layer and the backward LSTM layer at corresponding moments.

**Fig. 5:**
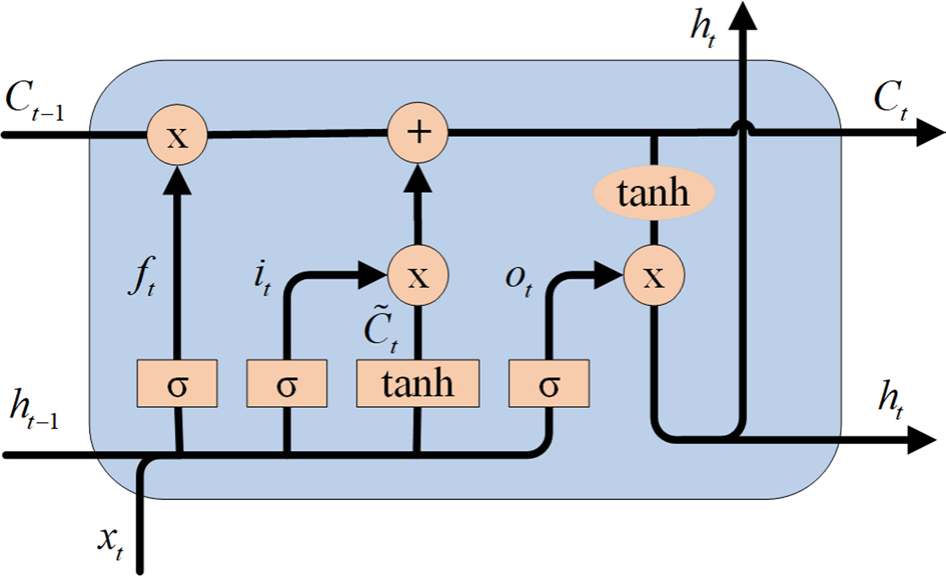
LSTM model diagram, which is controlled by three “gates”: the forgetting gate *f*_*t*_, the input gate *i*_*t*_, and the output gate *o*_*t*_. Besides, a set of memory units are employed to learn when to forget historical information and when to update

For basic LSTM structure, a set of memory units are employed to learn when to forget historical information and when to update, as shown in Fig. 5. At moment *t*, the memory unit *C*_*t*_ records all historical information up to the current moment, and it is also controlled by three “gates”: the forgetting gate *f*_*t*_, the input gate *i*_*t*_, and the output gate *o*_*t*_. The forgetting gate *f*_*t*_ determines what information to discard from the cellular state, as shown in (5). It views *h*_*t−*1_ (the previous hidden state) and *x*_*t*_ (the current input), then prints a number between 0 and 1 for each number in the state *C*_*t−*1_ (the previous state), with 1 being wholly retained and 0 being completely deleted.

5 $$f_{t} = \sigma (W_{f} \cdot [h_{t - 1} ,x_{t} ] + b_{f} )$$

The input gate determines what information is stored in the cellular state. First, the input gate’s Sigmoid activation function determines which values we will update, as shown in (6).

6 $$i_{t} = \sigma (W_{i} \cdot [h_{t - 1} ,x_{t} ] + b_{i} )$$

Then, an activation function *tanh*() creates a candidate vector $\tilde{C}_{t}$(new information), which will be added to the cell state, as shown in (7). Finally, combine the two vectors to create the updated value.

7 $$\tilde{C}_{t} = \tanh (W_{c} \cdot [h_{t - 1} ,x_{t} ] + b_{c} )$$

Update the last status value *C*_*t−*1_ to *C*_*t*_. Multiply the previous state value *C*_*t−*1_ by *f*_*t*_ to indicate what we expect to forget. Then add the obtained value $i_{t} * \tilde{C}_{t}$ and get the new state value *C*_*t*_, as shown in (8).

8 $$C_{t} = f_{t} * C_{t - 1} + i_{t} * \tilde{C}_{t}$$

The output gate determines what to output, and this output will be based on the current cell state. First, a Sigmoid activation function is used to determine which parts of the cell state we want to output, as shown in (9).

9 $$o_{t} = \sigma (W_{o} \cdot [h_{t - 1} ,x_{t} ] + b_{o} )$$

Then, the cell state is passed tanh to normalize the value between − 1 and 1 and multiplied by the output of the output gate to complete the output of which part of the information is determined by the output gate, as shown in (6).

10 $$h_{t} = o_{t} * {\text{tanh}}(C_{t} )$$

where *x*_*t*_ is the input of the time network. *f*_*t*_, *i*_*t*_ and *o*_*t*_ represent the states of forgetting gate, input gate, and output gate respectively. *W*_*f*_, *W*_*i*_, *W*_*c*_, *W*_*o*_ and *b*_*f*_, *b*_*i*_, *b*_*c*_, *b*_*o*_ represent weight matrix and deviation vector respectively.

LSTM has shown great advantages in modeling time series data due to its design characteristics, which can effectively solve long-term dependence and gradient disappearance existing in standard recurrent neural networks [36]. BiLSTM, by combining forward and backward LSTM, not only solves the gradient disappearance or gradient explosion problem but also fully considers the meaning of the current base fragment context [37].

### m^6^A site prediction based on BiLSTM

**Fig. 6:**
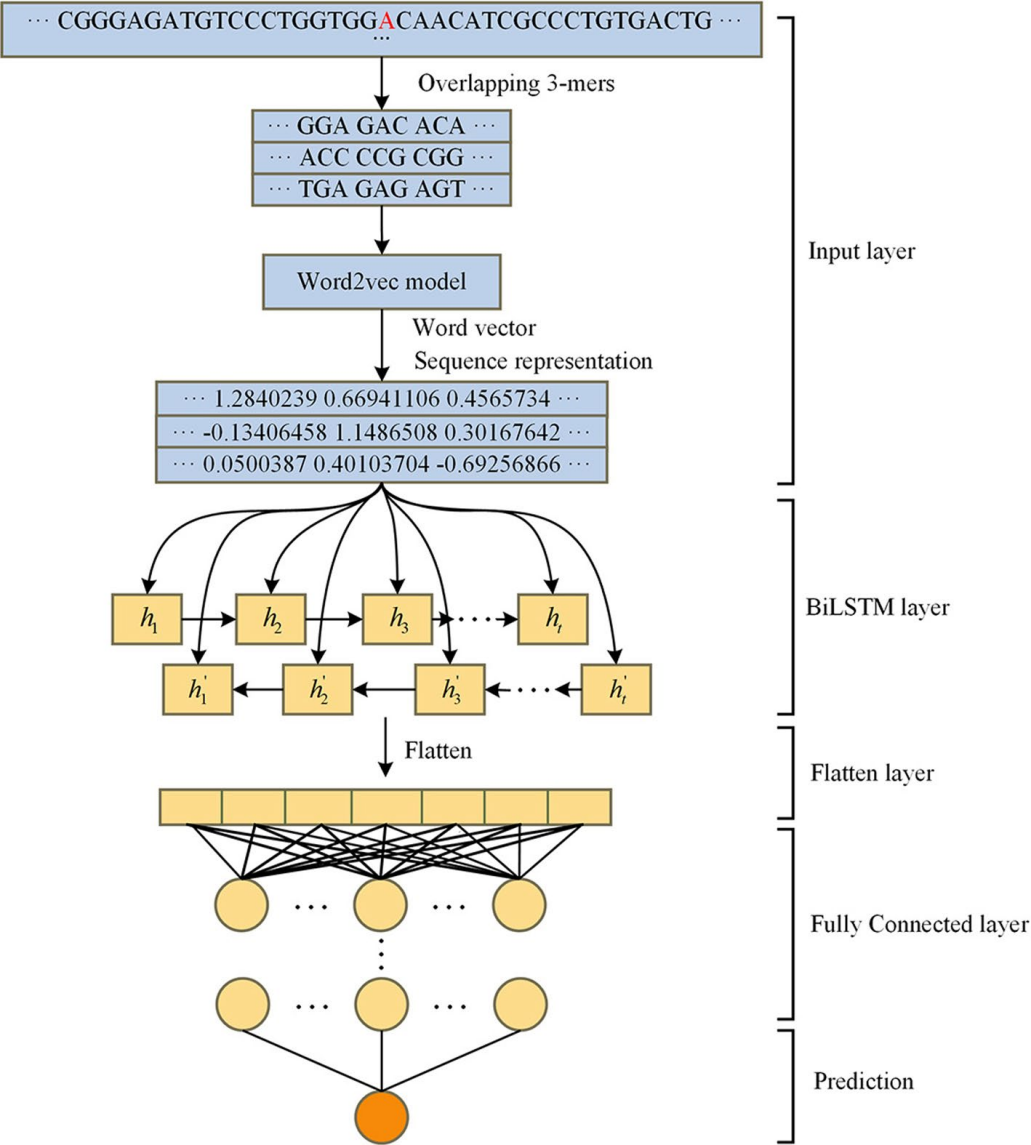
Predictors based on Word2vec and BiLSTM circular neural network, which adopts a five-layer architecture, including the input layer, BiLSTM layer, flattening layer, full connection layer, and prediction layer, among which the input layer handles data pre-processing

Three m^6^A site predictors are constructed by combining the BiLSTM and three sequence feature encoding methods, such as One-hot, RNA word embedding, and Word2vec respectively. Take Word2vec as an example, the predictor adopts a five-layer architecture, including the input layer, BiLSTM layer, flattening layer, full connection layer, and prediction layer, among which the input layer handles data pre-processing, as shown in Fig. 6.

The Word2vec model trains the pre-processed sequences, and the word vectors of each pseudo-RNA word are obtained to form a dictionary. Then, each sequence’s subsequence is represented by the corresponding word vector in the dictionary, and the feature matrix of 999 × 100 is finally obtained, which is exactly the input of the BiLSTM layer. BiLSTM has the memory capacity to learn the long-term context-dependence of sequence and extract the global features of sequences. To avoid overfitting, the dropout [38] module is adopted in the BiLSTM layer with the “drop” probability being 0.2. The data was then flattened into one dimension, followed by a full connection layer for final output. The full connection layer in Fig. 6 consists of three full connections, consisting of 256, 128, and 64 neurons, respectively, which helps to improve the complexity of the model. More full connection layers, the nonlinear expression ability of the model can be improved, such that the learning ability of the model is improved. Each neural is activated by ReLU [39] function, and dropout is also employed with 0.5 dropout probability. Finally, Sigmoid [40] defined in (11) is adopted to predict the probability of the existence of m^6^A sites in the given sequence.

11 $$\hat{y}(x) = sigmoid(x) = \frac{1}{{1 + e^{ - x} }}$$

### m^6^A site prediction based on ensemble integration

**Fig. 7:**
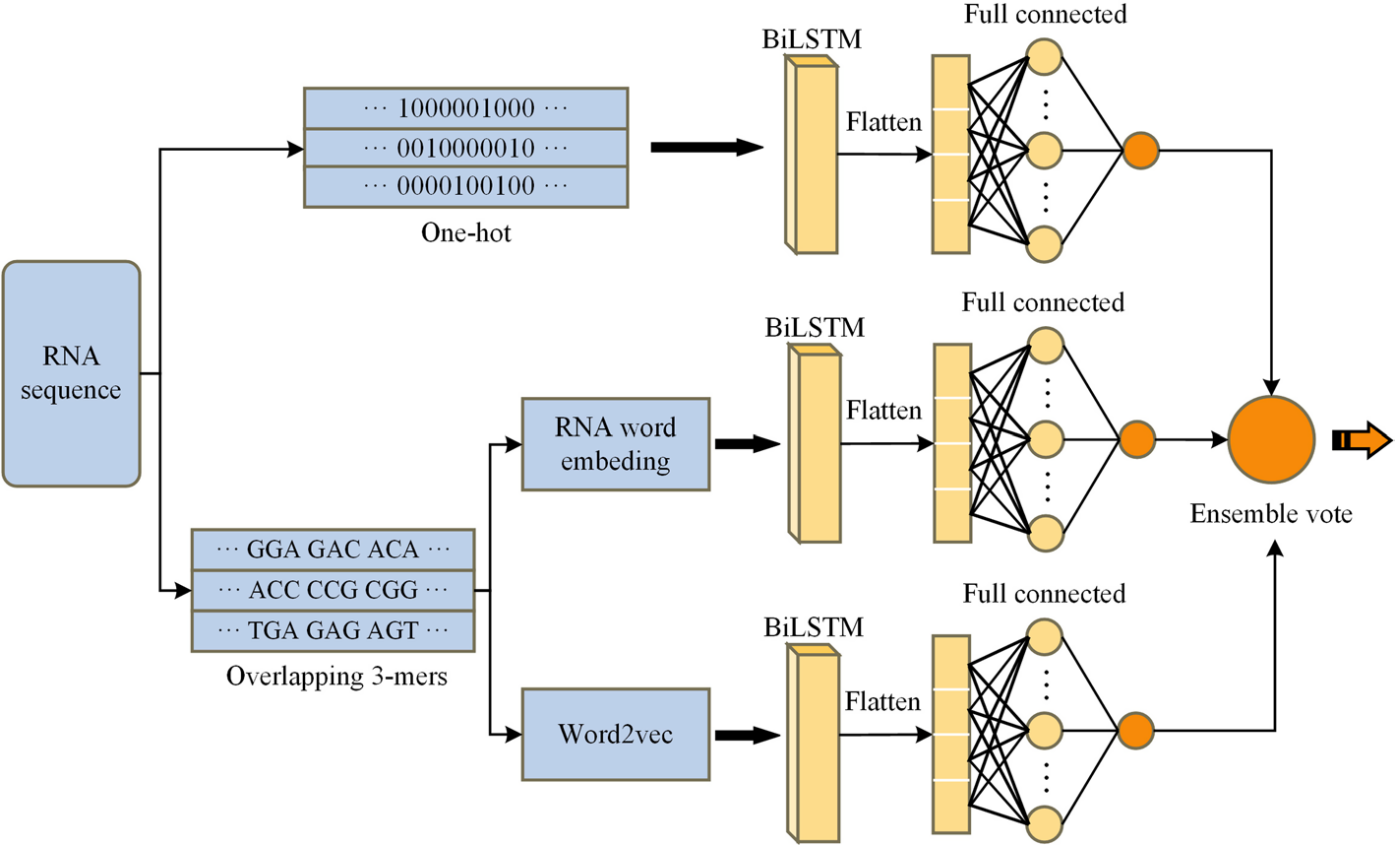
EDLm^6^APred model architecture. The figures showed the workflow of our method. The mRNA sequences were predicted by three different deep learning classifiers. Then they ensemble vote for the final results

As is known, different feature encoding method views sequence from different perspectives. One-hot and RNA word embedding describe the specific information of the RNA modification site in the sequence window. Word2vec utilizes an external neural network to thoroughly learn the semantic information between the context of the sequence. Thus, different predictors may take complementary effects on prediction performance. Therefore, an ensemble predictor named EDLm^6^APred based on One-hot, RNA word embedding, and Word2vec followed by BiLSTM is formulated, and the structure is shown in Fig. 7. With three predictors with different encodings, it aims to represent the sequences from more thorough perspectives. The weighted weights of the three predictors are obtained by the grid search method.

## Data Availability

The data supporting the findings of the article is available at the webserver www.xjtlu.edu.cn/biologicalsciences/EDLm6APred.

## Abbreviations

m^6^A: N^6^-methyladenosine
EDLm^6^APred: Ensemble deep learning approach for mRNA m^6^A site prediction
MeRIP-Seq: Methylated RNA immunoprecipitation sequencing
BiLSTM: Bi-directional long short-term memory
LSTM: Long short-term memory
RNN: Recurrent neural network
CNN: Convolutional Neural Network
NLP: Natural language processing
AUROC: Area under the receiver operation curve
ACC: Accuracy
MCC: Matthews correlation coefficient
